# Study of Hard Protein Corona on Lipid Surface of Composite Nanoconstruction

**DOI:** 10.3390/nano14211767

**Published:** 2024-11-04

**Authors:** Anna V. Epanchintseva, Svetlana V. Baranova, Julia E. Poletaeva, Irina A. Bakhno, Elena I. Ryabchikova, Ilya S. Dovydenko

**Affiliations:** Institute of Chemical Biology and Fundamental Medicine SB RAS, 630090 Novosibirsk, Russia; annaepanch@niboch.nsc.ru (A.V.E.); swb@niboch.nsc.ru (S.V.B.); poletaeva@niboch.nsc.ru (J.E.P.); bakhno@niboch.nsc.ru (I.A.B.)

**Keywords:** multilevel nanoconstructs, gold nanoparticles, hard protein corona, LC–MS/MS analysis, apolipoproteins

## Abstract

The composition of the protein corona covering any nanoparticle (NP) when it enters a biological fluid determines the parameters of the NP’s interaction with the body. To “control” these parameters, it is important to know the composition of the protein corona, the determination of which is a complex task associated with the two-layer organization of the corona (hard and soft coronas). In a previous publication, we reported obtaining lipid-coated NPs with a full protein corona, isolating them, and proving the presence of the corona on the surface of the NPs. This work reports on the preparation, isolation, and purification of lipid-coated NPs bearing a hard corona. The protein corona composition was determined by using the LC–MS/MS method. Thirty-seven serum proteins were identified with a high degree of reliability. The hard corona contained various apolipoproteins, including apolipoprotein E, which can potentially affect the penetration of NPs into the cell.

## 1. Introduction

Nanoparticles (NPs) are considered a promising means of targeted drug delivery for the treatment of various diseases, and the efforts of many researchers are aimed at developing ways to target them toward specific cells. However, when NPs enter the body, they immediately interact with components of biological fluids and adsorb them, mainly proteins, forming a “corona” on the surface of the nanoparticles [1,2]. In this way, the surface of the introduced NPs is modified, and as numerous studies show, the corona on the surface of the NPs determines the interaction of the NPs with the body’s cells [3,4,5]. Accordingly, in order to control the behavior of NPs introduced into the body, it is necessary to be able to determine and purposefully change the composition of the protein corona.

The task of determining the composition of the protein corona is sophisticated, and the basis for success involves reliable methods for the isolation of corona-bearing NPs, ensuring the safety of the corona and the possibility of analyzing of its proteins. The difficulty in preserving the protein corona on isolated NPs and the subsequent study of its composition are largely determined by the two-layered nature of the corona [2]. The first layer, called the hard corona, is firmly bound to the surface of the NPs and consists of proteins whose desorption time significantly exceeds the time of the experiments. The second layer is the soft corona, the proteins of which are in dynamic equilibrium with the environment. Its composition is extremely variable; therefore, in the process of NP isolation, it is impossible to preserve the soft corona in its native state.

Lipid nanoparticles (LNPs), which are the most widely used nanodrug carriers, occupy a special place in protein corona research. When LNPs come into contact with biological fluid, a protein corona is formed [6]; however, samples of LNPs bearing the corona are usually contaminated with endogenous biological nanoparticles (bio-NPs) [7]. The term “bio-NPs” integrates extracellular vesicles (EVs) and lipoprotein particles (LPs), both of which contain various proteins. It is obvious that contamination of engineered LNPs carrying a corona with bio-NPs will inevitably lead to distortion of the composition of the corona, and this leads to the emergence of an additional complex task in the workflow: the purification of LNP samples from bio-NPs.

Recently, we proposed a method for the photofixation of a complete protein corona, including the hard and soft coronas, on the surface of model LNPs [8]. We used previously developed and thoroughly studied multilevel nanoconstructs (MLNCs) as model LNPs [9]. In brief, the MLNC is a multilayer construct designed for siRNA delivery, in the center of which are gold nanoparticles (AuNPs) with siRNA molecules non-covalently adsorbed onto them. The non-covalent AuNP/siRNA complex is coated with a lipid envelope consisting of natural phospholipids and one ionizable lipidoid. The composition of the envelope is similar to the lipid composition of LNPs used for the delivery of therapeutic nucleic acids [10]. Despite the apparent complexity of the construct, in experiments on the isolation of MLNCs carrying a hard corona or a fixed full corona, it has been shown to be an ideal model for the isolation of corona-bearing NPs. Thus, the heavy core of MLNCs makes it possible to easily purify them of the unbound proteins of biological fluids and bio-NPs using low-speed centrifugation [8]. 

In this work, we continued to study the protein corona formed on the surface of MLNCs. We demonstrated the possibility of the desorption of hard corona proteins from the surface of MLNCs and studied the composition of a hard corona using LC–MS/MS. 

## 2. Materials and Methods

### 2.1. Preparation of the MLNC

The assembly of MLNCs involves three steps: the synthesis of AuNPs, the preparation of gold cores (AuNPs with non-covalently adsorbed siRNA molecules) and lipid films, and the encapsulation of the cores into a lipid envelope. 

AuNPs were obtained by the citrate reduction method using HAuCl_4_ (Aurat PAO, Moscow, Russia) [11]. In brief, 5 mL of 38.8 mM Na_3_C_6_H_5_O_7_ (Sigma–Aldrich, St. Louis, MO, USA) solution was added to 46 mL of a boiling 1.1 mM HAuCl_4_ solution with vigorous stirring. After 17 min, the boiling solution was removed from the heat, and the reaction mixture was left at room temperature for 24 h. The resulting AuNPs were filtered through a PTFE membrane with a pore diameter of 0.2 μm (Mdi, Camp Hill, PA, USA) and stored at 4 °C.

The cores were prepared according to [12], with minor modifications. Briefly, 4 mM of citrate-stabilized AuNPs at a concentration of 3.6 nM were incubated with 0.7 μM of siRNA for 22 h at room temperature in the presence of 5.6 mM NaCl (Honeywell, Seelze, Germany) and 0.1 mM MgSO_4_ (Honeywell, Seelze, Germany). After that, suspensions of the core NPs were centrifuged at 16,100× *g* for 40 min at 25 °C, and the supernatant was discarded.

Lipid films were prepared as described in [13]. A solution of 1 mM egg phosphatidylcholine (Avanti, Alabaster, AL, USA) and DOPE (Avanti, Alabaster, AL, USA) in a CHCl_3_/CH_3_OH mixture (Reachem, Moscow, Russia) (1:1) (90 μL) and a solution of 1 mM DOME2 (2-[[4-dodecylamino-6-oleylamino-1,3,5-triazine-2yl]-(2-hydroxyethyl)amino]ethanol) in CHCl_3_ (10 μL) were diluted in 1 ml of CHCl_3_ in a 10 mL round-bottom flask. The solvent was evaporated at 12 mmHg without thermostatting. To remove the residual solvent, the obtained lipid film was dried via vacuum in a desiccator; then, the flask with the film was kept at −18 °C for 16 h. All of the described steps were carried out in an argon atmosphere.

The procedure of complete MLNC assembly was described in detail in our previous publication [13]. The pellet containing the core NPs was diluted in water to a final volume of 900 μL and mixed with 31 μL of 10 mM NaH_2_PO_4_ (Reatex, Moscow, Russia) at pH 4.5. The resulting solution was added to the lipid film and sonicated for 15 min (25 °C, 90 W). After that, 69 μL of 0.01 M Na_2_HPO_4_ (Reatex, Moscow, Russia) was added to the mixture, followed by 10 μL of 1 mM stearic acid-conjugated peptide (Str-(RL)_4_G-NH_2_) (Alambion, Voronezh, Russia), and the mixture was sonicated for 5 min (25 °C, 90 W).

### 2.2. Incubation of MLNCs with 50% Solution of FBS

The resulting 2.5 nM MLNC suspensions were mixed with a 50% solution of FBS (Thermo Fisher Scientific, Waltham, MA, USA) in 2 mM of a phosphate buffer with a pH of 7.2 (Reatex, Moscow, Russia) (PB) in a volume ratio of 4 to 1 and incubated for 15 min at 25 °C. Purification of the MLNCs was carried out, in general, according to [8], with some modifications. The MLNCs incubated with FBS were subjected to centrifugation in 75% glycerol (Applichem, Darmstadt, Germany) containing 1 mM PB at 2000× *g* and 25 °C for 15 min. Red-colored fractions of the resulting MLNCs were collected and transferred to 15 mL tubes. The suspension was diluted 3.33 times with 1 mM PB and centrifuged at 3000× *g* and 25 °C for 10 min, and the supernatant was discarded. The pellets of the MLNCs were transferred to 1.5 mL tubes, concentrated by centrifugation at 2000× *g* and 25 °C for 10 min, joined into a single tube, and washed with 1 mL of 1 mM PB with subsequent centrifugation at 2000× *g* and 25 °C for 10 min. The washing process was repeated twice.

### 2.3. Electrophoresis 

Proteins bound to the MLNC surface were analyzed by sodium dodecyl sulfate-polyacrylamide gel electrophoresis using 7% PAAG under Laemmli conditions with subsequent staining using Coomassie brilliant blue.

### 2.4. Transmission Electron Microscopy 

A drop of a sample was adsorbed for 1 min on a pre-prepared copper grid; the excess liquid was removed by a pipette, then the grid was placed on a drop of 0.5% aqueous uranyl acetate solution for 10 s, and excess liquid was removed with filter paper. Grids were air dried and examined using a Jem1400 transmission electron microscope (Jeol, Tokyo, Japan). Digital images were collected with a Veleta digital camera (EM SIS, Münster, Germany).

### 2.5. Digestion of FBS and HC/MLNC with Trypsin

The procedure of digestion by trypsin was carried out according to the manufacturer’s protocol using the trypsin enhancer ProteaseMAX™ surfactant (Promega, Madison, WI, USA), with some modifications. For digestion, we used 4 μL of 4% FBS or 5–10 μL HC/MLNC; hereinafter, this is designated as a sample. One volume of a sample was mixed with 2 μL of 1% surfactant (Promega, Madison, WI, USA) in a 50 mM ammonium bicarbonate buffer with pH 7.8 and incubated for 1 min at room temperature. To eliminate the MLNCs from the HC/MLNC sample, a centrifugation step at 12,000× *g* for 30 min at room temperature was performed. Then, the supernatant was collected in a clean tube and diluted by a 50 mM bicarbonate buffer with pH 7.8 to 87.74 μL, and then 1 μL of 0.5 M DTT (Molekula GmbH, München, Germany) was added. The resulting mixture was incubated for 20 min at a temperature of 56 °C.

After restoration of the S-S bonds, a sample was incubated with 2.7 μL of 0.55 M iodoacetamide (Shanghai Macklin Biochemical Technology, Shanghai, China) at room temperature in the dark for 20 min. Digestion was completed by adding 7.56 μL (1.8 μg) of trypsin (Promega, Madison, WI, USA) to 1 μL of 1% surfactant. Proteolysis was then carried out for 3 h at 37 °C with shaking at 800 rpm. Trypsinolysis was stopped by adding trifluoroacetic acid (Sigma–Aldrich, St. Louis, MO, USA) to a final concentration of 0.5% and cooling the sample for 1 min on ice. The peptide mixture was centrifuged for 10 min at 12,000× *g* at room temperature and transferred to vials for further analysis.

### 2.6. LC–MS/MS Analysis

The LC–MS/MS analysis was performed at the Core Facility of Mass Spectrometric Analysis of ICBFM SB RAS. The trypsin-digested peptides were analyzed on an Orbitrap Q Ex-298 active HF high-resolution mass spectrometer (Thermo Fisher Scientific, Waltham, MA, USA) in the 200–1600 Da range, with separation conducted on a column by Poroshell 120 EC-C18 (Agilent Tech, Waldbronn, Germany). The peptides were washed with 2% phase B (0.1% formic acid and 100% acetonitrile) for 3 min at a flow rate of 250 mL/min and then eluted with gradient B from 2% to 40% for 8 min and another gradient B from 40% to 95% for 25 min. The spray voltage was set to 4.2 kV, and the normalized collision energy was set to 30% for MS/MS. Data-dependent ion selection was performed by using the most abundant 10 ions from a full MS scan for MS/MS analysis.

Peptide and protein identification were carried out using the Proteome Discoverer program (version 3.1, Thermo Fischer Scientific, Waltham, MA, USA) with the SEQUEST algorithm. The search was completed in the *Bos taurus* database, which was obtained from the Uniprot database (https://www.uniprot.org/) and the reviewed SwissProt database on 23 November 2023. The following settings were applied: an MS error tolerance of 10 ppm, an MS/MS error tolerance of 0.02 Da, trypsin as the protease, oxidation (M) as a variable modification, carbamidomethyl (C) as a fixed modification, and a peptide confidence level of medium.

## 3. Results

**Figure 1:**
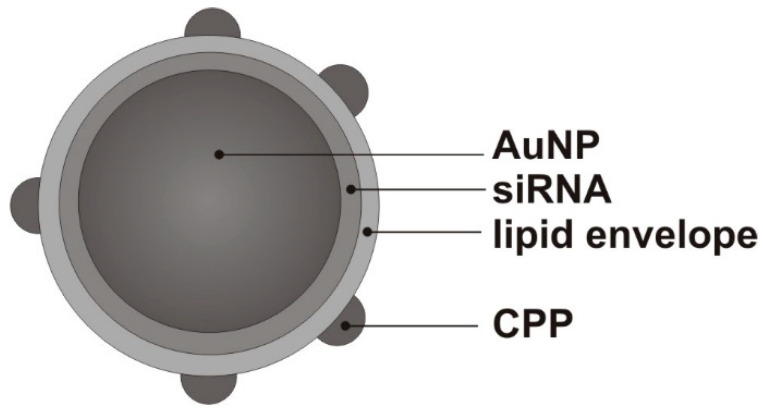
A schematic illustration of a multilevel nanoconstruct (MLNC): the gold core (AuNP) is covered with non-covalently bound siRNA; the lipid envelope is doped with a cell-penetrating peptide.

We showed earlier that developed MLNCs can be effectively used to isolate the hard and full protein corona formed on a lipid surface [8]. The presence of heavy nano-sized gold cores inside MLNCs makes it possible to use low-speed centrifugation, which allows for the gentle purification of the MLNCs from unbound proteins and other components of the biological environment, both in relation to the particles and the resulting protein corona (Figure 1).

According to published data, the binding time of hard corona proteins significantly exceeds the time of the experiment. During this time, proteins can change their conformation, which is assumed in the case of interactions of proteins with hydrophobic surfaces [14]. These interactions make it difficult for proteases to cleave hard corona proteins into peptides for subsequent mass analysis because part of the protein surface is shielded by the particle on which this protein is adsorbed and by other corona proteins, and because of possible change in its structure. To conduct the most complete analysis of the composition of a hard corona, it is necessary to carry out efficient cleavage of the corona, which requires the desorption of proteins from the surface of the particle, as well as relaxation of the protein structure due to the restoration of disulfide bridges [15].

### 3.1. Selection of Sample Preparation Conditions for Mass Analysis

This was the first time that we performed a hard corona assay on MLNCs, so we decided to separately study the effect of each component used in the standard trypsinolysis method on MLNC stability. It is advisable to conduct such studies in order to take into account the possible destruction of the MLNCs’ lipid envelopes at the time of implementation of the method, which may be accompanied by the release of gold cores that are capable of irreversibly binding proteins [16] and, accordingly, can result in data loss during mass analysis.

The trypsinolysis method [15] is based on the treatment of a protein mixture with a surfactant, followed by the restoration of disulfide bridges. Trypsin treatment is carried out after the protein structure has been weakened and sites for protease attack have become available. However, the use of a surfactant may damage the lipid envelope of MLNCs, and this will release the AuNPs hidden inside. In addition, dithiothreitol (DTT), which is used to reduce disulfide bridges, can covalently crosslink proteins with AuNPs. In this regard, we decided to check how the completeness of the obtained mass analysis data would change when using a standard method with the exclusion of one of the sample preparation stages. 

We prepared, under the previously defined conditions [8], samples of MLNCs that carried hard corona proteins on their surfaces. The washed and concentrated HC/MLNC pellets were processed in one of three ways: (i) HC/MLNCs were incubated with the surfactant; (ii) HC/MLNCs were treated with DTT, and in both cases (i) and (ii), MLNCs were then pelleted by centrifugation and the supernatant was trypsinized; and (iii) we used the method outlined in [15]. The mass analysis data of the obtained samples are presented in Table 1. The table includes proteins for which at least two peptides were identified.

The results obtained showed that the use of the surfactant only yielded a greater number of identifiable proteins compared to the method using DTT. Therefore, the use of the surfactant does not damage the lipid envelopes of MLNCs and does not result in the release of AuNPs. However, albumin contains many disulfide bridges, which were present after the treatment of the HC/MLNC sample with DTT and were not detected after surfactant treatment. The use of a standard technique combining the surfactant and DTT resulted in protein detection rates comparable to those of the surfactant-only method. The results obtained show that the use of the standard method does not damage the lipid envelope of the MLNC and allows for the identification of proteins detected using DTT or the surfactant. In subsequent studies, we used the standard method of sample preparation before trypsinolysis to determine the composition of the solid crown.

### 3.2. Mass Analysis of Proteins of the Hard Corona of MLNCs 

The protein composition of the hard corona formed on the surface of the MLNCs was analyzed in three independent experiments. For each experiment, MLNCs were prepared and incubated with 10% fetal bovine serum (FBS), and the obtained HC/MLNCs were purified to remove unbound proteins. The HC/MLNCs were then trypsinized using a standard method and the resulting peptide mixture was analyzed by LC–MS/MS. Each sample was analyzed in triplicate, with the intact 10% FBS used as a reference sample. In subsequent comparative analyses, proteins for which at least two peptides were identified were taken into consideration. An additional condition for the analysis was the presence of an abundance, the application of which reduced the number of proteins included in the analysis for HC/MLNCs from 60 to 38, and for the intact serum sample from 107 to 53. The complete data are presented in Table S1 (for the FBS analysis) and Table S2 (for the HC/MLNC analysis).

As expected, the majority of proteins belonging to the hard corona overlapped with proteins identified in the serum (Tables S1 and S2). At the same time, proteins that were not detected in the serum were found in the hard corona of MLNCs: apolipoprotein D and aggrecan core protein (Tables S1 and S2). Apparently, their concentration in the serum was so low that they could not be detected among the rest of the proteins. Moreover, serum proteins were not uniformly adsorbed onto the surface of the MLNCs, and after all of the procedures of isolation and washing, we obtained a sample that was artificially enriched with proteins included in the hard corona, which allowed us to identify minor components of the serum.

### 3.3. Distribution of Proteins of the Hard Corona of MLNCs by Mass

**Figure 2:**
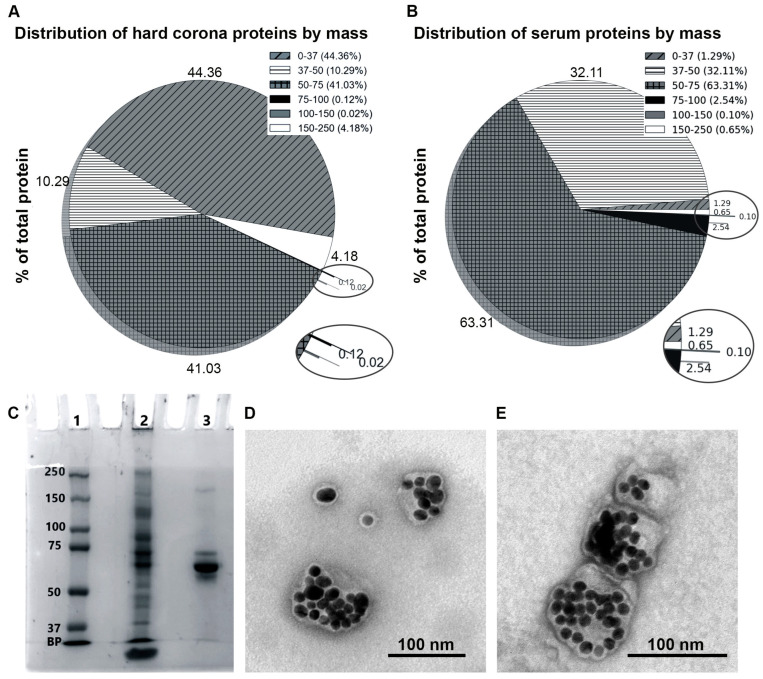
(**A**)—A pie chart showing the distribution of hard corona proteins by their masses; (**B**)—a pie chart showing the distribution of serum proteins by their masses; (**C**)—SDS-PAGE scanned image: 1—protein molecular weight marker, 2—HC/MLNCs, and 3—serum; (**D**)—a TEM image of an intact MLNC; (**E**)—a TEM image of an HC/MLNC using negative staining with 0.5% uranyl acetate.

The mass distribution of proteins present in the serum and adsorbed on the surface of the MLNCs varied significantly. Thus, the light protein fraction (up to 37 kDa) was predominantly adsorbed onto the lipid surface of MLNCs. This fraction in the serum is 1.29%, while, on the surface of MLNCs, its share increases to 44.36% (Figure 2A,B).

Proteins with a mass range of 50–75 kDa are the next most represented fraction of corona proteins on the surface of the MLNCs, including the main protein of the hard corona—albumin (the share of albumin is 22.42%). In contrast, this fraction of proteins in the intact serum is the main one, and the proportion of albumin is 61.72%. Next, in the hard corona, in descending order is the fraction of 37–50 kDa (10.29%), which is three times less than in the intact serum. Hard corona proteins with a mass of 75 to 150 kDa are represented by tenths of a percent in the composition of the hard corona; this fraction of proteins is also insignificant in the intact serum. At the same time, enrichment of up to 4.18% of the largest proteins (up to 250 kDa) is observed on the surface of the MLNCs, whereas in serum, their share does not exceed one percent. 

We observed a similar distribution of proteins on the electropherogram: the bulk of proteins were distributed in a range up to 75 kDa (Figure 2C). We were unable to visualize a hard corona on an MLNC in TEM, most likely because the corona is composed mainly of low mass proteins that are not large enough to form visible structures on the MLNC particle surface in TEM images (Figure 2D,E).

### 3.4. Distribution of MLNC Hard Corona Proteins by Isoelectric Point

The surfaces of native MLNCs carry a positive charge due to the presence of cell-penetrating peptides (CPPs) in the lipid envelope which carry four arginine residues. CPPs are present in abundance on the surface of MLNCs (10 mol% of the amount of lipids used); therefore, we assume that the electrostatic interaction due to its positive charge contributes comparably to other types of interactions to the formation of a hard corona [17]. Previously, we showed that the introduction of CPPs onto the surface of MLNCs changes their ζ-potential, the value of which is −37.5 ± 6.7 mV in 1 mM phosphate buffer (pH 7.4) [13]. 

This negative potential value is due to the electrostatic interaction of the positively charged arginine residues in CPPs with the phosphate anions of the solution surrounding the MLNCs. The fact that the ionic composition of the medium influences the charge of the MLNCs suggests that in the serum, the positively charged surface of the MLNCs will bind preferentially to negatively charged proteins.

**Figure 3:**
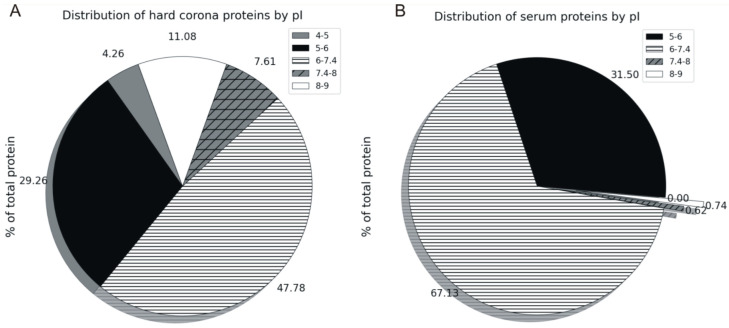
(**A**)—A pie chart showing the distribution of hard corona proteins by their isoelectric point; (**B**)—a pie chart showing the distribution of serum proteins by their isoelectric point.

To test this assumption, we assessed the distribution of proteins in the hard corona by the value of their isoelectric point (pI, Figure 3A).

In our data categorization, the threshold value pI = 7.4 was introduced intentionally since the serum solution for the MLNC incubation was prepared in phosphate buffer with pH = 7.4. We indirectly estimated the charge of proteins adsorbed on MLNCs.

The majority of hard corona proteins have a pI value less than 7.4 (the total charge is negative) and constitute 81.31%. Again, we noted the enrichment of minor serum proteins in the hard corona, detecting proteins that have a pI in the range of 4–5 in the hard corona, while proteins of this type were not observed in the intact serum (Figure 3A,B).

**Figure 4:**
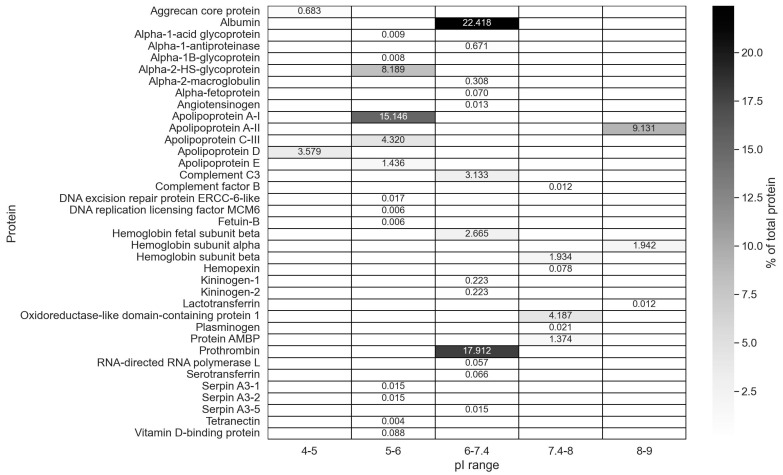
A heat map of the distribution of corona proteins by the value of their isoelectric point.

The proportion of proteins with pI = 7.4–9 (total positive charge) was increased in the hard corona relative to the serum. The presence of these proteins in the hard corona may be due to the fact that they interact with the MLNC surface non-electrostatically or have an affinity for already sorbed negatively charged proteins. The distribution of hard corona proteins by isoelectric point is shown in Figure 4.

The following negatively charged proteins, which are quantitatively predominant in the hard corona, have a pI lower than the pH of the serum solution used: albumin, prothrombin, apolipoprotein A-I, alpha-2-HS-glycoprotein, apolipoprotein C-III, apolipoprotein D, complement C3, and hemoglobin subunit beta. The majority of the positively charged protein fraction consists of apolipoprotein A-II and oxidoreductase-like domain-containing protein 1. Apolipoprotein A-II represents about half of the proteins with pI 7.4-9 (total positive charge). It has previously been suggested that this protein interacts with the lipid surface via the apolar lipid-binding surface of its amphipathic α-helices [18]. This suggests that the positively charged proteins of hard coronas on the surface of the MLNC interact with the particle surface without the involvement of electrostatic forces.

### 3.5. Distribution of Proteins of MLNC Hard Corona by Participation in Organism Functions

**Figure 5:**
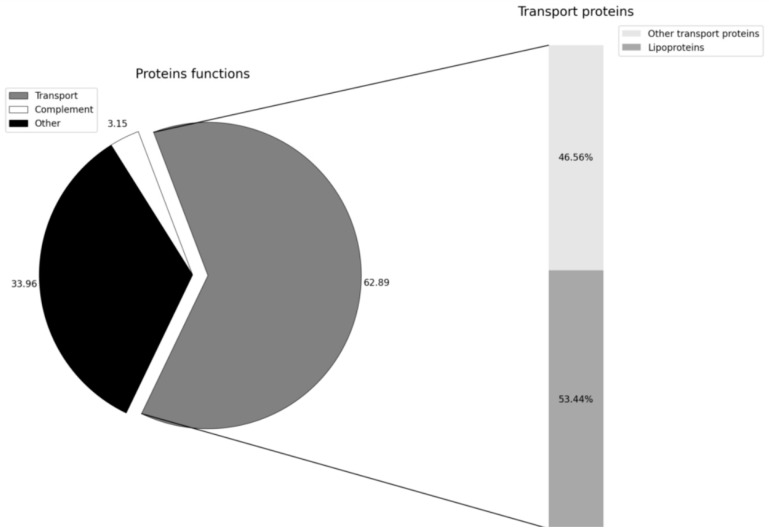
The pie chart shows the distribution of HC/MLNC proteins by their involvement in organism functions. The bar chart shows the proportions of apolipoproteins and other transport proteins.

It was interesting to know whether the hard corona on the MLNC surface contains transport proteins and proteins involved in immune reactions because such proteins can influence the distribution and elimination of nanoparticles in the body. The distribution of hard corona proteins is shown in Figure 5.

Proteins involved in immune reactions are represented by proteins of the complement system (complement C3 and complement factor B) and make up 3.15% of the total amount of hard corona proteins (Figure 4 and Figure 5; Table S1). The fraction of transport proteins is 62.89%, half of which are apolipoproteins. 

Synthetic lipid nanoparticles are similar in size and density to bio-NPs in biological fluids, and therefore, there is a possibility for their co-isolation. In this case, the results of the protein corona composition studies would be distorted, as reported in [7]. The heavy and dense core consisting of AuNPs allows for the isolation of the HC/MLNC without its contamination by bio-NPs. Nevertheless, when using our method to obtain full-corona MLNCs, we observed the presence of bio-NPs, which were a component of the soft corona [8].

**Figure 6:**
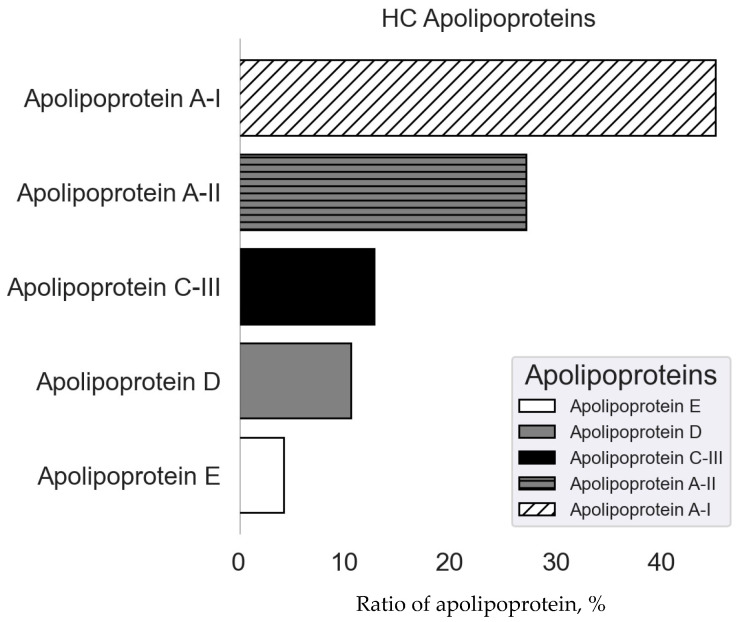
Ratio of lipoproteins in composition of HC/MLNCs.

The results obtained in this work show that all five types of detected apolipoproteins are part of the hard corona (Figure 6) and make up a third of all its proteins.

The major apolipoproteins are apolipoprotein A-I and apolipoprotein A-II, which account for more than 40% and 20% of the total apolipoproteins, respectively. Apolipoprotein C-III, apolipoprotein D, and apolipoprotein E were also found in the hard corona. Apolipoproteins detected in the hard corona (Table 2) are capable of binding to chylomicrons and lipoproteins of varying densities [19]. Among all apolipoproteins that make up the hard corona of MLNCs, it is worth noting apolipoprotein E, which is capable of binding to the low-density lipoprotein receptor on the surface of cells and, thereby, influencing the distribution of particles in the body.

## 4. Discussion

Previously, we demonstrated the possibility to isolate MLNCs bearing a complete protein corona under very gentle conditions [8]. In this work, we tested the possibility of determining the composition of the hard corona by tandem mass spectroscopy using the MLNC-FBS experimental system. The changes in the surface of MLNCs after contact with 10% FBS and subsequent purification were confirmed by electrophoresis and dynamic light scattering, and the presence of protein components was demonstrated using denaturing gel electrophoresis according to Laemmli. We did not expect the MLNC envelope would be so resistant to sample preparation reagents for mass analysis that we would obtain better results using the standard HC/MLNC sample preparation method prior to trypsinolysis than with the reagent-limited variants.

The obtained mass analysis data showed that the sorption of serum components on the surface of the MLNC was selective (Table S1, Figure 2A,B and Figure 3A,B). We detected minor serum components in the hard corona that could not be detected in the serum itself. This does not mean that these components were not present in the intact FBS and that what we saw were artifacts or contamination. This means that the concentration of these proteins in the serum is less than the detection limit and, due to their selective sorption on the surface of the MLNCs, their concentration in the HC/MLNC preparation increased and became suitable for further mass analysis. We observed a similar situation in gel electrophoresis: in the serum, only major components were visualized, most likely albumin and globulins, whereas in the HC/MLNC preparation, we observed a set of products in a wide range of masses (Figure 2C).

According to the obtained mass analysis data, the majority of proteins sorbed onto MLNCs do not exceed 75 kDa in mass, and half of them are in a range of up to 37 kDa. This observation helps explain why we were unable to visualize the hard corona on the surface of MLNCs in the TEM images, although the proteins were detected both in the gel after electrophoretic separation and by mass spectrometry. In our opinion, it was the small size of the MLNC hard corona proteins that prevented us from visualizing the corona, in contrast to the well-visualized hard corona on polymer NPs [20,21].

As noted above, the composition of the NP surface will further determine the composition of the protein corona, which determines the important role of NP surface functionalization in the formation of the protein corona. The surface of the MLNCs was functionalized with cationic CPPs. This functionalization was carried out to increase the efficiency of MLNC penetration into the cell and to overcome endosomal arrest. The obtained results show that this type of functionalization does not prevent the formation of a protein corona [8] and determines the type of adsorbed proteins.

As we can see, 80% of all corona proteins had a negative total charge based on the fact that their pI was less than the pH value of the environment. A major part of this pool of proteins includes albumin, prothrombin, alpha-2-HS-glycoprotein, and many different apolipoproteins, which make up a third of the corona. Of the five types of apolipoproteins in the MLNC’s hard corona, apolipoprotein E is of the most interest because this apolipoprotein can bind to receptors such as LDLR and SR-BI on the surface of hepatocytes and other cells in the body [19].

This property of apolipoprotein E has been used to create nanoparticle-based delivery systems. It has been shown that nanoparticles modified with apolipoprotein E, or carrying it as part of a protein corona, are capable of penetrating into various tissues, such as a human prostate cancer cell line (PC3) [22], a mouse brain endothelioma cell line (bEnd3) [23], and mouse brain cells [24]. Moreover, it has been shown that particles carrying apolipoprotein E on their surface are able to overcome the blood–brain barrier [25,26], which “fuels” interest in obtaining particles of covalently/non-covalently bound apolipoprotein E [27]. 

In previous studies, we showed that the transfection of cells with MLNCs in the presence of 10% FBS lead to the successful delivery of siRNA cargo, which was confirmed by changes in the expression of the reporter protein. This fact suggests that the corona formed around the MLNC during transfection does not prevent the penetration of particles into the cell [12].

## 5. Conclusions

In this work, we demonstrated that our previously proposed model of lipid NPs, which is based on MLNCs, for studying the formation of a protein corona on the surface of a lipid envelope can be used to determine the composition of a hard corona using tandem mass spectroscopy LC–MS/MS. Using this approach, we determined the composition of the hard corona of an MLNC in which 37 serum proteins were identified with a high degree of reliability. The hard corona contained various apolipoproteins, including apolipoprotein E, which can potentially affect the penetration of NPs into the cell.

The practical aspect of our study is that the MLNC-based system that we developed is suitable for examining the formation and composition of protein coronas on lipid surfaces. The proposed approach, published earlier [8] and presented in this paper, allows us to obtain purified particles bearing a hard protein corona on their surface, the proteins of which can be analyzed by tandem mass spectrometry. The developed method can be further applied to the study of the composition of the protein corona formed on lipid surfaces that contain cationic lipids.

## Figures and Tables

**Table 1:** Differences in the composition of the hard corona on MLNCs depending on the trypsinolysis conditions.

No.	Protein	Number of Identified Peptides in Sample Treated		
		With DTT	With Surfactant	By Standard Way
0	Albumin	5	-	28
1	Alpha-2-HS-glycoprotein	-	-	8
2	Apolipoprotein A-I	-	21	14
3	Apolipoprotein A-II	-	6	4
4	Apolipoprotein C-III	-	2	3
5	Apolipoprotein D	-	-	6
6	Apolipoprotein E	-	-	3
7	Complement C3	-	-	22
8	Complement factor B	-	6	-
9	Hemoglobin fetal subunit beta	-	-	5
10	Hemoglobin subunit alpha	3	6	6
11	Keratin, type I cytoskeletal 10	-	4	-
12	Keratin, type I cytoskeletal 17	-	3	-
13	Keratin, type I cytoskeletal 28	-	2	-
14	Keratin, type II cytoskeletal 7	-	4	-
15	Keratin, type II cytoskeletal 78	-	2	-
16	Oxidoreductase-like domain-containing protein 1	-	2	-
17	Pigment epithelium-derived factor	-	7	-
18	Protein AMBP	-	-	3
19	Prothrombin	-	-	9
20	Tetranectin	2	3	-

**Table 2:** The presence of apolipoproteins in the MLNC’s hard corona and their ability to bind with bio-NPs.

Apolipoprotein\Type of Bio-NP	CM	HDL	VLDL
Apolipoprotein A-I	+	+	
Apolipoprotein A-II	+	+	
Apolipoprotein C-III	+	+	+
Apolipoprotein D	+		
Apolipoprotein E	+	+	+

CM—chylomicron; HDL—high-density lipoprotein; VLDL—very low-density lipoprotein.

## Data Availability

The data are available on request from the corresponding authors.

## Supplementary Materials

The following supporting information can be downloaded at: https://www.mdpi.com/article/10.3390/nano14211767/s1, Table S1: Results of tandem mass spectrometry analysis for FBS; Table S2: Results of tandem mass spectrometry analysis for HC/MLNC.
